# Monosodium Glutamate Induces Cellular Stress, Endoplasmic Reticulum Stress, Mitochondrial Dysfunction, and Cell Death in Intestinal Epithelial Cells

**DOI:** 10.1111/all.70007

**Published:** 2025-08-21

**Authors:** Bingjie Zhao, Huseyn Babayev, Can Zeyneloglu, Yagiz Pat, Duygu Yazici, Sena Ardicli, Asunción García‐Sánchez, Oliva Giannelli Viscardi, Mübeccel Akdis, Kari C. Nadeau, Cezmi A. Akdis, Ismail Ogulur

**Affiliations:** ^1^ Swiss Institute of Allergy and Asthma Research (SIAF) University of Zurich Davos Switzerland; ^2^ Department of Genetics Faculty of Veterinary Medicine, Bursa Uludag University Bursa Turkey; ^3^ Department of Biomedical and Diagnostic Science, School of Medicine University of Salamanca Salamanca Spain; ^4^ Department of Environmental Health Harvard T.H. Chan School of Public Health Boston Massachusetts USA

**Keywords:** apoptosis, epithelium, inflammation

To the Editor,

1

The epithelial barrier theory suggests that persistent periepithelial inflammation plays a key role in aggravating inflammatory diseases. Such inflammation can be triggered by environmental toxic substances that compromise the epithelial barrier, including food additives, personal hygiene and cleaning products, air pollution, and nano‐ and microplastics [1]. Monosodium glutamate (MSG) is a frequently used food flavor enhancer linked to obesity, metabolic dysregulation, oxidative stress, irritable bowel syndrome, and early‐onset rectal cancer [2, 3, 4]. Some research has also shown that glutamate‐mediated excitotoxicity is related to autism [5]. Commonly used recommended levels are 0.1%–1%. MSG is sometimes present in foods on its own, while sometimes it appears in combination with disodium guanylate (DSG) and disodium inosinate (DSI), synergistically enhancing the umami flavor (Table S1). Although animal studies have demonstrated the effects of these flavor enhancers, data on their impact on human tissues remain limited [4, 6].

Caco‐2 cells and gut‐on‐a‐chips were used in our study to test the effects of these taste enhancers on gut epithelial barrier. Caco‐2 cells in gut‐on‐a‐chip form a tubular 3D structure with an intact epithelial barrier similar to physiological and pathological conditions that allows the assessment of barrier integrity [7]. Our results show that MSG, DSG, DSI, and their combinations exhibited cytotoxicity in epithelial monolayers starting at 1% MSG, 0.5% DSG, 2% DSI, and 0.5% for the combined treatment of all three flavor enhancer substances (Figure 1A; Figure S1A–D). Transepithelial electrical resistance (TEER) significantly decreased after 1 day of exposure to 1% MSG and 2 days of exposure to 0.25% and 0.5% MSG (Figure 1B; Figure S2A). One‐day exposure to 1% DSG or the 1% combined treatment also reduced TEER, while even high concentrations of DSI did not have a similar effect (Figure 1B,C; Figure S2B–D). To define the short‐term effects of MSG and the combination of three taste enhancers, TEER measurements were taken at baseline, 5 min, 30 min, and hourly during the first 4 h. Significant decreases in TEER were observed after 4 h of 1% MSG exposure and after just 30 min with 1% exposure to their combination (Figure S3). In addition, after 3 days, 1% MSG and the combined treatment significantly increased paracellular flux, indicating compromised epithelial barrier integrity (Figure 1D; Figure S4A–D). At 1% MSG and the exposure to their combination, the occludin staining showed a decrease in staining intensity. Additionally, morphological changes characterized by irregular and heterogeneous staining with occludin were observed between 0.5% and 1% concentrations of MSG and 0.25% and 1% of the combined treatment (Figure S5). Moreover, we evaluated the effect of glutamic acid on these models and observed cytotoxicity starting at a concentration of 1%. TEER significantly decreased after a 1‐day exposure to 1% glutamic acid, whereas paracellular flux showed a tendency to increase after 3 days of exposure (Figure S6). After 24 h, exposures to 1% MSG, 1% DSI, and the combined treatment significantly increased reactive oxygen species (ROS) levels (Figure 1E). ROS levels increased by 1% MSG were significantly reduced by an antioxidant, N‐acetyl‐L‐cysteine (NAC) (Figure 1F).

**FIGURE 1:**
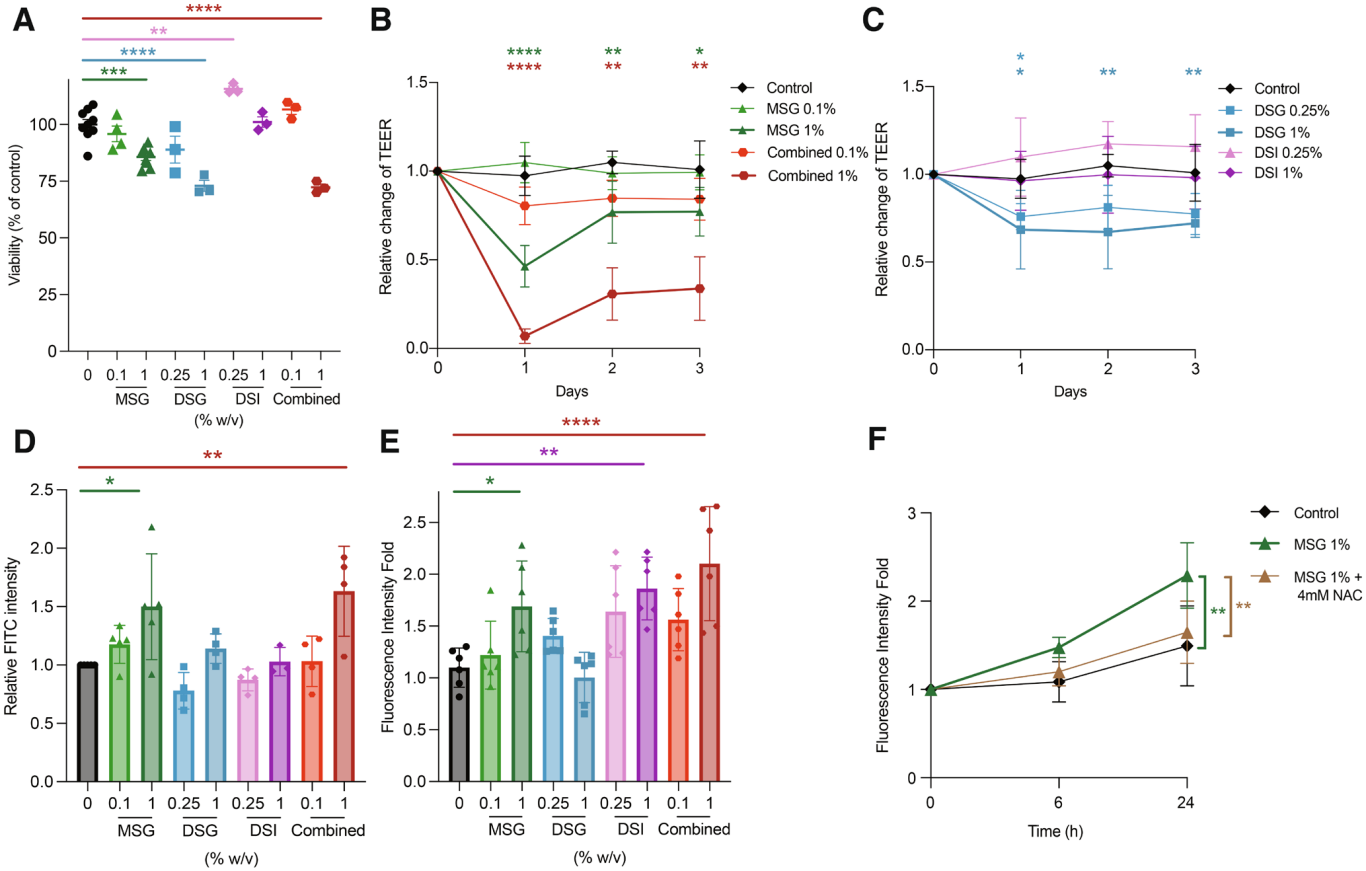
Effects of MSG, DSG, DSI, and their combination on intestinal epithelial cell viability, barrier integrity, and oxidative stress. (A) The MTT assay shows relative cell viability expressed as a percentage of the untreated control after 24 h of exposure to increasing concentrations of MSG, DSG, DSI, and their combined treatment. Statistical significance is indicated by asterisks (*p < 0.05, **p < 0.01, ***p < 0.001, ****p < 0.0001 by one‐way ANOVA). (B) The changes in transepithelial electrical resistance (TEER) relative to initial values are presented over time for cells exposed to 0.1% and 1% MSG and combined effect of three taste enhancers. (C) TEER measurements for cells treated with 0.25% and 1% DSG, as well as 0.25% and 1% DSI, are shown over a similar time course. Symbols represent mean ± SD of four to six independent experiments, and statistical significance relative to the control is indicated as above. (D) Paracellular flux (PF) measurements on day 3. Bars represent mean ± SD of four to six independent experiments, and significance is denoted above. (E) Intracellular reactive oxygen species (ROS) levels after 24 h of exposure to 1% MSG, 1% DSG, 1% DSI, and the combined treatment demonstrate the presence of oxidative stress. Bars represent fold changes ± SD of three independent experiments, and asterisks mark statistically significant differences from the control. (F) ROS levels measured over time following exposure to 1% MSG in the presence or absence of 4 mM N‐acetyl‐L‐cysteine (NAC) illustrate a significant attenuation of oxidative stress. Symbols represent mean ± SD of three independent experiments, and significance is indicated as above. DSG, disodium guanylate; DSI, disodium inosinate; MSG, monosodium glutamate. [Correction added on 3 September 2025, after first online publication: Figure 1 is replaced with a new figure.]

RNA‐sequencing analysis demonstrated that 1% MSG and the combined treatment caused distinct shifts in molecular pathways (Figure 2A), with a notable increase in the number of differentially expressed genes (Figure 2B). We found that 1% MSG affected cell growth and differentiation, signaling pathways, stress responses, transport processes, mitochondrial processes, and autophagy (Figure 2C). The combined doses of MSG, DSG, and DSI (the combined treatment) amplified the effects predominantly caused by MSG (Figure 2C). At the translational level, both 1% MSG and the combined treatment led to an upregulated oxidative stress response, endoplasmic reticulum (ER) stress, which includes genes involved with protein folding defects (Figure 2D,E, Tables S2 and S3). The downregulated pathways were primarily associated with mitochondrial dysfunction (Figure 2F, Table S4). Additionally, macroautophagy and regulation of autophagy were other affected pathways after exposure to 1% MSG and the combined treatment (Figure 2G). Increased expression of *Deptor*, a component of the mTORC1 complex, was observed alongside enhanced TORC1 signaling, which aligns with previous studies [8]. Genes involved in autophagy, such as *SMURF1*, *PINK1*, *ULK1*, and *MAP1LC3B2*, were upregulated following exposure to 1% MSG and the combined treatment (Figure 2G, Table S5). Given that autophagy is integral to proteostasis, we speculate that impaired autophagy may have disrupted proteostasis, contributing to the accumulation of unfolded proteins and subsequent ER stress [9]. The combined treatment largely mirrored and enhanced the effects observed with MSG alone, indicating that MSG may be the principal driver of these changes.

**FIGURE 2:**
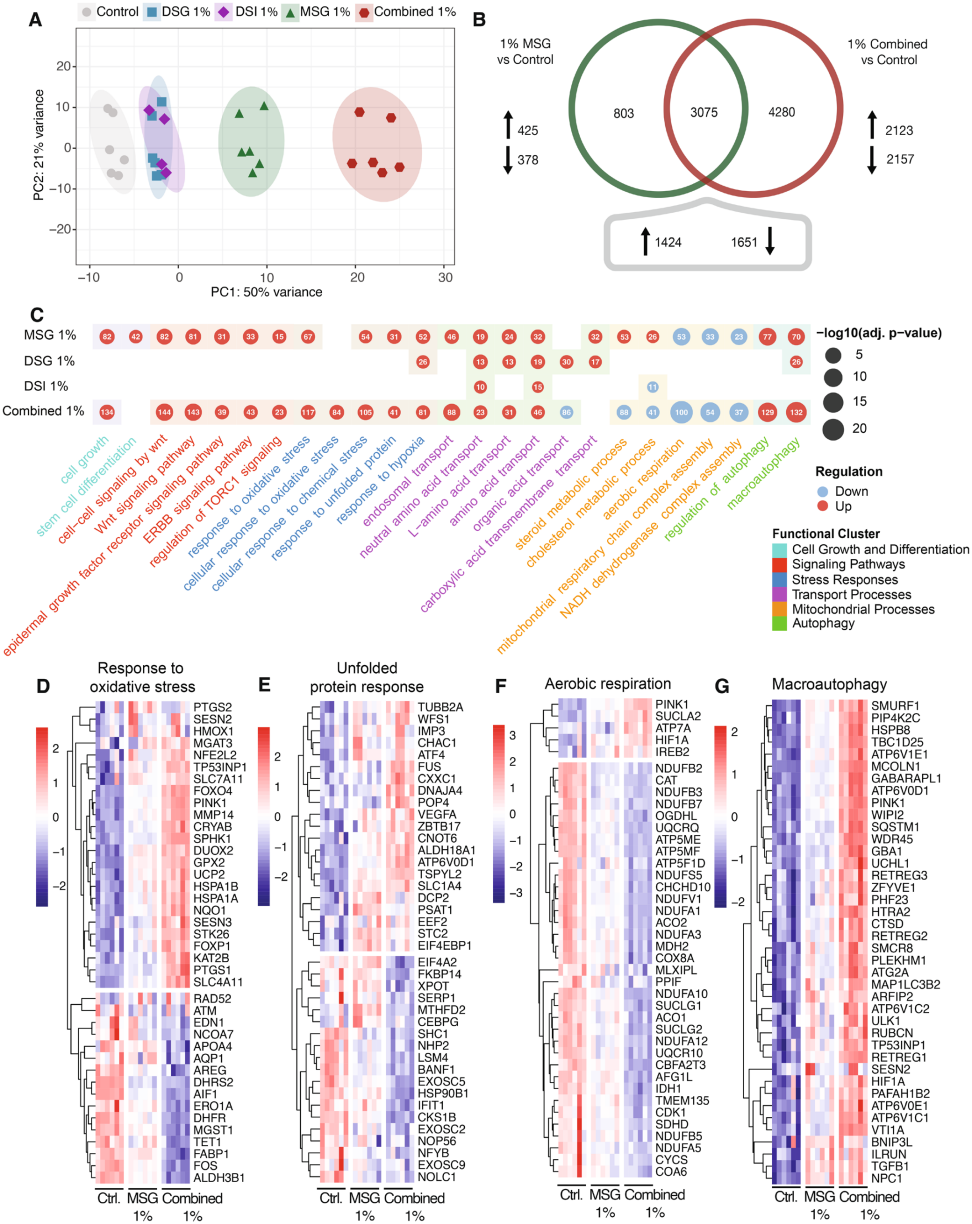
Differential gene expression and pathway perturbations induced by MSG and the combined taste enhancer exposure. (A) Principal component analysis (PCA) of RNA‐seq data from Caco‐2 cells treated with 1% MSG, 1% DSG, 1% DSI and 1% combined treatment, or control reveals distinct clustering of treated groups n: 6, illustrating global changes in gene expression profiles induced by these additives. (B) The Venn diagram illustrates a set of genes that changed expression under 1% MSG and 1% combined treatment exposure, with 1424 genes being upregulated and 1651 genes downregulated. (C) Enrichment analysis identifies pathways prominently affected by MSG and the combined treatment. Notable among these are those governing responses to cell growth and differentiation, signaling pathways, stress responses, transport processes, mitochondrial processes and autophagy. (D–G) Heatmaps depict relative expression levels of representative genes involved in oxidative stress responses, unfolded protein responses, aerobic respiration, and macroautophagy comparing controls, cells exposed to 1% MSG, and those exposed to the combined treatment. Red and blue colors indicate upregulation and downregulation, respectively, while clustering patterns show that MSG and combined treatments disrupt proteostasis, enhance oxidative and ER stress responses, and compromise mitochondrial function.

In conclusion, our findings suggest that under daily exposure conditions, MSG, DSG, DSI, and their combination disrupt the epithelial barrier integrity, induce various destructive biological processes, including oxidative stress, unfolded protein response, and mitochondrial dysfunction, as well as altered key molecular pathways governing autophagy and proteostasis in intestinal epithelial cells. Additionally, our study shows that NAC mitigates the oxidative stress, which is induced by 1% MSG in intestinal epithelial cells. Future research will explore antioxidant strategies to reduce MSG‐induced cell damage. While caution is needed in extrapolating these results directly to human health, this work underscores the importance of further research on the molecular mechanisms by which common dietary food additives influence gut barrier function and cellular homeostasis.

## Author Contributions

B.Z., C.A.A., and I.O. conceived and designed the study. B.Z., H.B., C.A.A., and I.O. planned the experiments. H.B. helped with the bioinformatics and graphics. C.Z., Y.P., D.Y., S.A., A.G.S., and O.G.V. helped with the experiments. B.Z., H.B., and I.O. wrote the manuscript. M.A., K.C.N., and C.A.A. reviewed the manuscript.

## Conflicts of Interest

M.A. has received research grants from the Swiss National Science Foundation, Bern; research grant from Stanford University; Leading House for the Latin American Region, Seed Money Grant. She is the Scientific Advisory Board member of Stanford University Sean Parker Asthma Allergy Center, CA; Advisory Board member of LEO Foundation Skin Immunology Research Center, Copenhagen; and Scientific Co‐Chair of World Allergy Congress (WAC) Istanbul, 2022, Scientific Program Committee Chair, EAACI. K.C.N. currently reports grants from National Institute of Allergy and Infectious Diseases (NIAID), National Heart, Lung, and Blood Institute (NHLBI), National Institute of Environmental Health Sciences (NIEHS); Stock options from IgGenix, Seed Health, ClostraBio, Cour, Alladapt; Consultant for Excellergy, Red tree ventures, Regeneron, and IgGenix; Co‐founder of Alladapt, Latitude, and IgGenix; National Scientific Committee member at Immune Tolerance Network (ITN), and National Institutes of Health (NIH) clinical research centers; patents include, “Mixed allergen composition and methods for using the same,” “Granulocyte‐based methods for detecting and monitoring immune system disorders,” and “Methods and Assays for Detecting and Quantifying Pure Subpopulations of White Blood Cells in Immune System Disorders.” C.A.A. has received research grants from the Swiss National Science Foundation, European Union (EU CURE, EU Syn‐Air‐G), Novartis Research Institutes (Basel, Switzerland), Stanford University (Redwood City, Calif), Seed Health (Boston, USA) and SciBase (Stockholm, Sweden); is the Co‐Chair for EAACI Guidelines on Environmental Science in Allergic diseases and Asthma; is on the Advisory Boards of Sanofi/Regeneron (Bern, Switzerland, New York, USA), Stanford University Sean Parker Asthma Allergy Center (CA, USA), Novartis (Basel, Switzerland), Glaxo Smith Kline (Zurich, Switzerland), Bristol‐Myers Squibb (New York, USA), Seed Health (Boston, USA), and SciBase (Stockholm, Sweden); and is the Editor‐in‐Chief of Allergy. I.O. is chair of the EAACI Epithelial Cell Biology Working Group. B.Z., H.B., C.Z., Y.P., D.Y., and S.A. declare no relevant conflicts of interest. B.Z. reports grant from the CSC scholarship program of China (No. 2023).

## Supporting information


**Data S1.** Supporting Information

## Data Availability

The data that support the findings of this study are available from the corresponding author upon reasonable request.
