# Supplementary material for: Monosodium Glutamate Induces Cellular Stress, Endoplasmic Reticulum Stress, Mitochondrial Dysfunction, and Cell Death in Intestinal Epithelial Cells
Source: Allergy. 2025 Aug 21;80(10):2916–20. doi: 10.1111/all.70007 (PMC12486344; doi:10.1111/all.70007)
Supplement: Supplementary file 1 — Data S1. Supporting Information [file ALL-80-2916-s001.pdf]

Monosodium glutamate induces cellular stress, endoplasmic reticulum stress, mitochondrial dysfunction and cell death in intestinal epithelial cells

Bingjie Zhao[1], Huseyn Babayev[1], Can Zeyneloglu[1], Yagiz Pat[1], Duygu Yazici[1], Sena Ardicli[1], Asunción García-Sánchez[1,2], Oliva Giannelli Viscardi[1], Mübeccel Akdis[1], Kari C. Nadeau[3], Cezmi A. Akdis[1], Ismail Ogulur[1]

1. Swiss Institute of Allergy and Asthma Research (SIAF), University of Zurich, Davos, Switzerland
2. Department of Biomedical and Diagnostic Science, School of Medicine, University of Salamanca, Salamanca, Spain
3. Department of Environmental Health, Harvard T.H. Chan School of Public Health, Boston, Massachusetts, USA.

Corresponding authors:

Ismail Ogulur, Swiss Institute of Allergy and Asthma Research (SIAF), University of Zurich, Herman-Burchard-Strasse 9, CH-7265 Davos Wolfgang, Switzerland, e-mail: ismail.ogulur@siaf.uzh.ch

Cezmi A. Akdis, Swiss Institute of Allergy and Asthma Research (SIAF), University of Zurich, Herman-Burchard-Strasse 9, CH-7265 Davos Wolfgang, Switzerland, e-mail: akdisac@siaf.uzh.ch

Supplementary Methods

Cell cultures

The Caco-2 cell line (American Type Culture Collection, Manassas, VA, USA) was routinely maintained in 75-cm^2 cell culture flasks at 37˚C in a humidified atmosphere with 5% CO_2. Cells were grown in a culture medium composed of DMEM (Gibco-BRL, Invitrogen, Carlsbad, Calif) with 1% penicillin/streptomycin (Sigma-Aldrich, Saint Louis, USA), 1% non-essential amino acids (Sigma-Aldrich), 1% sodium pyruvate (Sigma-Aldrich), 10% fetal bovine serum (Sigma-Aldrich), and up to 70-90% confluency. The media was changed three times a week.

OrganoPlates (Mimetas, NL) were used for investigating the effects of food flavour enhancer exposures. The plates consisted of 64 chips with Caco-2 tubules. While incubating, perfusion was done by placing the plates on an interval rocker (OrganoFlow, Mimetas, NL) set at a 7° inclination and 8-minute interval inside an incubator. Cells in chip were grown in a culture medium composed of EMEM (ATCC, Manassas, USA) with 1% penicillin/streptomycin (Sigma-Aldrich, Saint Louis, USA), 1% non-essential amino acids (Sigma-Aldrich), 1% sodium pyruvate (Sigma-Aldrich), 10% fetal bovine serum (Sigma-Aldrich). The media was changed every 2-3 days.

Food flavor enhancer exposures

Monolayer cells were treated for one day for cytotoxicity, and organ-on-a-chip cultures were treated on the apical compartments for three days with DMEM containing 1%, 0.5%, 0.25% and 0.1% (w/v) of monosodium glutamate (MSG), disodium guanylate (DSG), disodium inosinate (DSI) or the combined treatment.

We ordered MSG and DSG (Thermo Scientific Chemicals, catolog numbers: A12919.30 and 226600250, Zug, Switzerland), DSI (Sigma-Aldrich, cat: 352195-40-5, St. Louis, USA),

L(+)-Glutamic acid (Thermo Scientific Chemicals, cat: 156212500) and N-Acetyl-L-cysteine (Sigma-Aldrich, cat:A9165). To exclude the endotoxin influence on barrier function, endotoxin assays were performed at different dilutions of food flavor enhancers. Endotoxins were not found at detectable levels (< 0.01 EU/ml) across all the dilutions.

Cytotoxicity measurement using MTT assay

Cell viability was assessed using the MTT assay (Invitrogen, USA) following the manufacturer's instructions. Briefly, 2×10^4 cells were seeded in a 96-well plate and incubated under standard culture conditions. The cells were then treated in different conditions. The MTT reagent (thiazolyl blue tetrazolium bromide) was added to each well and incubated at 37°C for 4 hours, allowing viable cells to reduce MTT to insoluble formazan crystals via mitochondrial dehydrogenase activity. The formazan was then dissolved in a solubilization solution (e.g., DMSO), and absorbance was measured at 540–570 nm using a microplate reader. The absorbance values directly indicate cell viability. Data for MSG cultures were obtained from three independent experiments, each conducted with a minimum of three replicates. For DSG, DSI and combined cultures, three replicates were used per condition.

Transepithelial electrical resistance on Caco-2 grown on organ-on-a-chip models

TEER measures electrical resistance across epithelial/endothelial monolayers ($\Omega\cdot cm^2$), reflecting tight junction integrity and paracellular permeability. High TEER values (e.g., 500–1,100 $\Omega\cdot cm^2$ in mature intestinal models) indicate intact barriers, while reduced values signal compromised integrity.[1] For OrganoPlates, after refreshing the media, baseline TEER measurement was performed using OrganoTEER (Mimetas, NL). Gradient concentrations of MSG, DSG and DSI (1%, 0.5%, 0.25%, 0.1% and 0.05%) were added to the apical side of the chips and TEER values were measured after 24h, 48h and 72h. The effect of each sample was also expressed as the TEER relative to baseline values. Each culture was normalized against a

control and calculated from four to six independent experiments, each performed with at least four replicates.

Paracellular permeability assay

Paracellular permeability was assessed by measuring the apical-to-basolateral flux of FITC-labelled 4-kDa dextran (Sigma-Aldrich). Briefly, after exposure with food flavor enhancers for 72 hours, 2 mg/ml of dextran was added to the apical side of the lumen of the Caco-2 tubules and incubated at 37° C for 24 hours. The samples (100 μl) were harvested from the basolateral side of the tubules and the amount of passaged dextran was evaluated with an ELISA reader (Mithra LB 940; Berthold Technologies, Bad Wildbad, Germany) at 480 nm. Each culture was normalized against a control and calculated from four to six separate experiments, each of which was performed in at least triplicates.

RNA-sequencing

Total RNAs were isolated using an RNeasy Plus Micro Kit (Qiagen, Hilden, Germany) according to the manufacturer's protocol. RNA-seq was performed with the TruSeq Stranded mRNA Sample Prep Kit (Illumina, San Diego, Calif) on the Illumina NovaSeq 6000 in Functional Genomic Center of Zurich (FGCZ). For RNA-seq analysis, six biological replicates were included per condition from a single experiment.

Statistical analysis

All statistical analyses and associated figures were generated with R package and GraphPad Prism software (version 10.2.0; GraphPad Software, La Jolla, Calif). Differences among multiple groups were evaluated using one-way ANOVA, followed by Dunnett’s multiple comparions test. Differences among groups with different effectors including conditions and times were evaluated using RM two-way ANOVA with Geisser-Greenhouse

correction, followed by Dunnett's multiple comparions test. Differences were considered statistically significant when P < .05.

Supplementary Table 1. Foods which contain MSG or MSG, DSG and DSI together

Food Category	Foods Containing MSG	Foods Containing MSG, DSG and DSI together
**Processed Meats**	Bacon, pepperoni	Sausages, lunch meats, beef jerky, hot dogs, meat snacks
**Fast Foods**	Fast food and chain restaurant dishes, such as noodles, stir-fries, soups	Chicken nuggets
**Canned Goods**	Canned vegetables	Canned soups
**Frozen Foods**	Frozen breakfasts, frozen dinners	Frozen pizzas
**Fermented or Aged Foods**	Aged cheeses (like Parmesan and Roquefort)	Fermented bean paste
**Snacks**	Tortilla chips, flavored popcorn, mixed snacks	Potato chips, flavored crackers
**Condiments**	Barbecue sauce, ketchup, mayonnaise, salad dressings	Soy sauce
**Seasoning Blends**	Soup mixes, stew mixes	Meat marinades, vegetable seasonings
**Instant Foods**	Instant soup mixes	Instant noodle
**Dairy Products**	Skim and low-fat milk (with MSG-containing powdered milk), yogurt, ice cream	Energy drinks
**Naturally Foods**	Tomatoes, seaweed, peanuts, broccoli, peas, walnuts	Fish, mushrooms[2]

Data are from Kurihara K et al. [2] and websites.[3,4]

Supplementary Table 2. Differentially expressed genes in oxidative stress response

Gene Name	Function Summary
**Upregulated genes**	
PTGS2	Encodes cyclooxygenase-2, an enzyme involved in converting arachidonic acid to prostaglandin H2, a precursor for various prostaglandins. It plays a key role in inflammation and is a target for NSAIDs.
SESN2	Encodes sestrin-2, a protein involved in regulating cell growth, metabolism, and survival under stress conditions. It inhibits mTORC1 activity and is involved in autophagy and oxidative stress responses.
HMOX1	Encodes heme oxygenase-1, an enzyme that catalyzes the degradation of heme into biliverdin, carbon monoxide, and ferrous iron. It plays a protective role against oxidative stress and inflammation.
MGAT3	Encodes mannosyl (Beta-1,4-Mannosyl-Glycoprotein 4-Beta-N-Acetylglucosaminyltransferase), an enzyme involved in glycoprotein synthesis and modification.
NFE2L2	Encodes nuclear factor erythroid 2-related factor 2, a transcription factor that regulates antioxidant and detoxification responses. It is crucial for protecting against oxidative stress and promoting cellular homeostasis.
TP53INP1	Encodes tumor protein p53-inducible nuclear protein 1, involved in regulating autophagy and cellular stress responses. It is linked to cancer and metabolic disorders.
SLC7A11	Encodes solute carrier family 7 member 11, a protein involved in the transport of cystine and glutamate across cell membranes. It plays a role in oxidative stress response and ferroptosis regulation.
FOXO4	Encodes forkhead box O4, a transcription factor involved in regulating cellular processes such as growth and differentiation and also plays a role in oxidative stress and insulin signaling.
PINK1	Encodes a protein which is a mitochondrial serine/threonine kinase involved in mitochondrial quality control and Parkinson's disease.
MMP14	Encodes matrix metalloproteinase 14, an enzyme involved in the degradation of the extracellular matrix. It plays roles in tissue remodeling and cell migration.
CRYAB	Encodes alpha-crystallin B chain, a small heat shock protein involved in protecting against protein misfolding.
SPHK1	Encodes sphingosine kinase 1, an enzyme that phosphorylates sphingosine to produce sphingosine-1-phosphate, a signaling molecule involved in TNF-alpha signaling and the NF-kappa-B activation pathway important in cell survival, migration, inflammatory, antiapoptotic, and immune processes.
DUOX2	Encodes dual oxidase 2, an enzyme involved in the production of hydrogen peroxide for thyroid hormone synthesis and antimicrobial defense.
GPX2	Encodes glutathione peroxidase 2, an enzyme that protects cells from oxidative damage by reducing hydrogen peroxide and lipid hydroperoxides.
UCP2	Encodes uncoupling protein 2, involved in regulating mitochondrial membrane potential and reactive oxygen species production.
HSPA1B	Encodes heat shock protein family A (Hsp70) member 1B, a chaperone protein involved in protein quality control and stress response.
HSPA1A	Encodes heat shock protein family A (Hsp70) member 1A, similar to HSPA1B, involved in protein folding and stress response.
NQO1	Encodes NAD(P)H quinone dehydrogenase 1, an enzyme that protects against oxidative stress by reducing quinones and maintaining cellular redox balance.
SESN3	Similar to SESN2, involved in regulating cell growth, metabolism, and stress responses through mTORC1 inhibition.
STK26	Encodes serine/threonine kinase 26, involved in regulating cell growth, differentiation, apoptosis, metabolism and immune regulation.
FOXP1	Encodes forkhead box P1, a transcription factor involved in regulating development, immune responses, and cancer progression.
KAT2B	Encodes lysine acetyltransferase 2B, involved in cell growth and transcriptional regulation.
PTGS1	Encodes cyclooxygenase-1, similar to PTGS2 but constitutively expressed in many tissues, involved in promoting cell proliferation during tumor progression
SLC4A11	Encodes solute carrier family 4 member 11, involved in ion transport across cell membranes and implicated in corneal endothelial dystrophy.
**Downregulated genes**	
RAD52	Encodes RAD52 homolog, involved in DNA repair mechanisms, particularly homologous recombination.
ATM	Encodes ataxia-telangiectasia mutated, a kinase involved in DNA damage response and repair, including double-strand breaks.
EDN1	Encodes endothelin-1, a potent vasoconstrictor peptide involved in cardiovascular regulation and implicated in hypertension.
NCOA7	Encodes nuclear receptor coactivator 7, involved in transcriptional regulation by RNA polymerase II.
APOA4	Encodes apolipoprotein A-IV, involved in lipid metabolism.
AQP1	Encodes aquaporin-1, a water channel protein involved in water transport across cell membranes.
AREG	Encodes a growth factor involved in promoting normal epithelial cells and suppressing the proliferation of specific aggressive carcinoma cell lines.
DHRS2	Encodes dehydrogenase/reductase 2, involved in the metabolism of various compounds.
AIF1	Encodes allograft inflammatory factor 1, involved in immune responses and promoting macrophage activation.
ERO1A	Encodes endoplasmic reticulum oxidoreductase 1 alpha, involved in protein folding and disulfide bond formation in the endoplasmic reticulum.
DHFR	Encodes dihydrofolate reductase, an enzyme involved in folate metabolism.

MGST1	Encodes microsomal glutathione S-transferase 1, involved in detoxification processes and protection against oxidative stress.
TET1	Encodes ten-eleven translocation 1, involved in DNA demethylation and epigenetic regulation.
FABP1	Encodes fatty acid-binding protein 1, involved in lipid metabolism and transport within cells.
FOS	Encodes Fos proto-oncogene, a transcription factor involved in cell proliferation, differentiation, and transformation.
ALDH3B1	Encodes aldehyde dehydrogenase 3 family member B1, involved in detoxification processes produced by alcohol metabolism and lipid peroxidation and is important in protection from oxidative stress.

Supplementary Table 3. Differentially expressed genes in unfolded protein response

Gene Name	Function Summary
**Upregulated genes**	
TUBB2A	Encodes a beta-tubulin protein, a component of microtubules involved in cell division, and intracellular transport. Defects are associated with brain malformations like cortical dysplasia.
WFS1	Encodes wolframin, a protein regulating calcium levels in cells, particularly in the endoplasmic reticulum. It is crucial for protein processing and cell signaling. Mutations are linked to Wolfram syndrome.
IMP3	Encodes U3 Small Nucleolar Ribonucleoprotein 3 involved in cell growth and differentiation.
CHAC1	Encodes a protein involved in the regulation of oxidative stress responses, unfolded protein response and glutathione level.
ATF4	Encodes a transcription factor activated during endoplasmic reticulum stress and mitochondrial stress which is involved in regulating gene expression related to stress responses and cellular adaptation.
FUS	Encodes a protein involved in RNA processing and transcriptional regulation.
CXXC1	Encodes a protein involved in transcriptional regulation affecting gene expression.
DNAJA4	Encodes a molecular chaperone involved in cell migration and protein refolding.
POP4	Encodes a ribonuclease involved in RNA processing and degradation, affecting gene expression and cellular homeostasis.
VEGFA	Encodes vascular endothelial growth factor A, a key regulator of angiogenesis and vascular development, implicated in cancer progression and tissue repair.
ZBTB17	Encodes a transcription factor involved in regulating gene expression during development and immune responses.
CNOT6	Encodes a component of the CCR4-NOT complex, involved in RNA degradation and transcriptional regulation, affecting gene expression and cellular homeostasis.
ALDH18A1	Encodes a protein involved in amino acid metabolism, particularly in the synthesis of proline and ornithine, affecting cellular energy metabolism.
ATP6V0D1	Encodes a part of the V-type ATPase complex, essential for maintaining cellular pH homeostasis and vesicular transport.
TSPYL2	Encodes a protein involved in transcriptional regulation and chromatin remodeling, affecting cell-cycle progression and potentially implicated in cancer.
SLC1A4	Encodes a transporter involved in neutral amino acid transport across cell membranes affecting cellular metabolism.
DCP2	Encodes a protein involved in RNA processing and degradation, particularly in the context of microRNA regulation.
PSAT1	Encodes phosphoserine aminotransferase 1, an enzyme involved in amino acid metabolism.
EEF2	Encodes eukaryotic translation elongation factor 2, crucial for protein synthesis and cellular growth.
STC2	Encodes stanniocalcin 2, a protein involved in regulating calcium and phosphate homeostasis, affecting intestinal calcium and phosphate transport.
EIF4EBP1	Encodes a protein involved in regulating protein synthesis by binding to eukaryotic translation initiation factor 4E (eIF4E), affecting cellular growth and metabolism.
**Downregulated genes**	
EIF4A2	Encodes a helicase involved in cap recognition and translation initiation, affecting gene expression and cellular homeostasis.
FKBP14	Encodes a protein locates in endoplasmic reticulum which is involved in accelerating protein folding and stress response.
XPOT	Encodes exportin-T, involved in the export of tRNA from the nucleus to the cytoplasm, affecting protein synthesis and cellular homeostasis.
SERP1	Encodes a protein involved in regulating endoplasmic reticulum unfolded protein responses.
MTHFD2	Encodes a protein involved in folate metabolism and can form an enzyme-magnesium complex which will bind to NAD.
CEBPG	Encodes a transcription factor involved in regulating gene expression of immunoglobulin heavy chain.
SHC1	Encodes a protein involved in cell signaling pathways, particularly in regulating cell life span and response to reactive oxygen species.
NHP2	Encodes a protein involved in rRNA processing and ribonucleoprotein complex assembly.
LSM4	Encodes a protein involved in pre-mRNA splicing by mediating U4/U6 snRNP formation .
BANF1	Encodes a protein involved in mitotic nuclear assembly, chromatin organization, DNA damage response and gene expression.

EXOSC5	Encodes a component of the exosome complex involved in RNA degradation and processing.
HSP90B1	Encodes a molecular chaperone locates in melanosomes and the endoplasmic reticulum and involved in folding and stabilizing other proteins.
IFIT1	Encodes a protein involved in antiviral responses and immune regulation.
CKS1B	Encodes a protein interacts with the catalytic subunit of cyclin-dependent kinases and is crucial for their biological activity and involved in cell cycle regulation.
EXOSC2	Encodes a component of the cytosol and nuclear exosome complex, involved in RNA degradation and processing, affecting gene expression and cellular homeostasis.
NOP56	Encodes a protein involved in ribonucleoprotein complex assembly and RNA processing and regulating cell growth.
NFYB	Encodes a subunit of the sequence-specific heterotrimeric transcription factor NF-Y involved in regulating gene expression.
EXOSC9	Encodes a component of the exosome complex, involved in RNA degradation and processing.
NOLC1	Encodes a protein involved in nucleolar organization and ribosome biogenesis.

Supplementary Table 4. Differentially expressed genes in aerobic respiration

Gene Name	Function Summary
**Upregulated genes**	
PINK1	Encodes a mitochondrial serine/threonine kinase involved in mitochondrial quality control and is believed to safeguard cells against mitochondrial dysfunction caused by stress.
SUCLA2	Encodes the beta subunit of succinyl-CoA ligase, an enzyme in the citric acid cycle. It plays a critical role in respiratory electron transport.
ATP7A	Encodes a copper-transporting ATPase involved in maintaining copper homeostasis.
HIF1A	Encodes hypoxia-inducible factor 1-alpha, a transcription factor that regulates gene expression in response to low oxygen levels and involved in energy metabolism.
IREB2	Encodes iron regulatory protein 2, involved in regulating iron metabolism by binding to iron-responsive elements in mRNA.
**Downregulated genes**	
NDUFB2	Encodes a subunit of NADH:ubiquinone oxidoreductase (Complex I), a crucial enzyme in the mitochondrial electron transport chain.
CAT	Encodes catalase, a key antioxidant enzyme that decomposes hydrogen peroxide into water and oxygen, protecting cells from oxidative damage.
NDUFB3	Encodes another subunit of Complex I in the mitochondrial electron transport chain, essential for energy production.
NDUFB7	Encodes a subunit of Complex I locates in mitochondrial inner membrane, involved in the electron transport chain and ATP synthesis.
OGDHL	Encodes a protein which is similar to oxoglutarate dehydrogenase (OGDH) of the OGDH complex, involved in the citric acid cycle and suppressing cell proliferation and promoting apoptosis..
UQCRQ	Encodes a subunit of the cytochrome b-c1 complex (Complex III) in the mitochondrial electron transport chain.
ATP5ME	Encodes a subunit of ATP synthase, crucial for ATP production in mitochondria.
ATP5MF	Encodes a subunit of ATP synthase, involved in ATP synthesis during oxidative phosphorylation.
ATP5F1D	Encodes a subunit of ATP synthase, essential for ATP production in mitochondria.
NDUFS5	Encodes a subunit of Complex I in the mitochondrial electron transport chain, involved in energy production.
CHCHD10	Encodes a protein involved in mitochondrial function and dynamics and maintaining cristae morphology or regulating oxidative phosphorylation.
NDUFV1	Encodes a subunit of Complex I, crucial for electron transport and ATP synthesis.
NDUFA1	Encodes a subunit of Complex I, involved in the electron transport chain and energy metabolism.
ACO2	Encodes aconitase 2, an enzyme in the citric acid cycle that catalyzes the conversion of citrate to isocitrate.
NDUFA3	Encodes a subunit of Complex I, essential for electron transport and ATP synthesis.
MDH2	Encodes malate dehydrogenase 2, an enzyme in the citric acid cycle that catalyzes the conversion of malate to oxaloacetate.
COX8A	Encodes a subunit of cytochrome c oxidase (Complex IV) in the mitochondrial electron transport chain.
MLXIPL	Encodes a transcription factor involved in glucose metabolism.
PPIF	Encodes PPlase, a protein involved in mitochondrial permeability transition pore regulation and cell death.
NDUFA10	Encodes a subunit of Complex I, involved in electron transport and ATP synthesis.
SUCLG1	Encodes the alpha subunit of succinyl-CoA ligase, another version of the enzyme involved in the citric acid cycle.
ACO1	Encodes aconitase 1, an enzyme in the citric acid cycle that catalyzes the conversion of citrate to isocitrate in the cytosol.
SUCLG2	Encodes the beta subunit of succinyl-CoA ligase, similar to SUCLA2 but with specificity for GDP instead of ADP.
NDUFA12	Encodes a subunit of Complex I, essential for electron transport and ATP synthesis.
UQCR10	Encodes a subunit of Complex III in the mitochondrial electron transport chain.
CBFA2T3	Encodes a transcription factor involved in hematopoiesis and potentially in cancer progression.
AFG1L	Encodes a protein involved in mitochondrial function and dynamics.
IDH1	Encodes isocitrate dehydrogenase 1, an enzyme involved in the citric acid cycle and potentially implicated in cancer.

TMEM135	Encodes a protein involved in mitochondrial metabolism by affecting the balance between mitochondrial fusion and fission.
CDK1	Encodes cyclin-dependent kinase 1, a key regulator of the cell cycle, particularly during the G2/M phase transition.
SDHD	Encodes a subunit of succinate dehydrogenase (Complex II) in the mitochondrial electron transport chain, also involved in tumor suppression.
NDUFB5	Encodes a subunit of Complex I, involved in electron transport and ATP synthesis.
NDUFA5	Also a subunit of Complex I, essential for electron transport and ATP synthesis.
CYCS	Encodes cytochrome c, a crucial electron carrier in the mitochondrial electron transport chain.
COA6	Encodes a protein involved in the assembly of Complex IV in the mitochondrial electron transport chain.

Supplementary Table 5. Differentially expressed genes in macroautophagy

Gene Name	Function Summary
SMURF1	Encodes an E3 ubiquitin ligase specific for receptor-regulated SMAD proteins in the bone morphogenetic protein (BMP) pathway. Involved in cell motility, cell signaling, and cell polarity
PIP4K2C	Encodes a phosphatidylinositol-4-phosphate-5-kinase involved in regulating mTORC1 signaling, autophagosome assembly and insulin receptor signaling. Plays a role in cardiac hypertrophy and fibrosis.
HSPB8	Encodes small heat shock protein which is involved in protein quality control and stress response, it also acts as a chaperone in partnership with Bag3, which promotes macroautophagy, often implicated in neuromuscular diseases.
TBC1D25	Encodes a protein which functions as a Rab GTPases, plays a role in facilitating the fusion of autophagosomes with endosomes and lysosomes
ATP6V1E1	Encodes a protein which encodes a part of the vacuolar ATPase complex, which is crucial for maintaining cellular pH homeostasis and vesicular transport.
MCOLN1	Encodes a member of the transient receptor potential (TRP) cation channel family, a protein locates in intracellular vesicular membranes, including lysosomes, and plays a role in the late endocytic pathway as well as in regulating lysosomal exocytosis.
GABARAPL1 (ATG8)	Encodes protein also known as ATG8 which facilitates Tat protein binding, phospholipid binding, and ubiquitin protein ligase binding. It is predicted to play a role in the cellular response to nitrogen starvation and macroautophagy. Found in the autophagosome and mitochondrion.
ATP6V0D1	Encodes the subunit of the V-type ATPase complex, essential for proton transport and cellular homeostasis.
PINK1	Encodes a mitochondrial serine/threonine kinase involved in mitochondrial quality control and Parkinson's disease. It is believed to safeguard cells against mitochondrial dysfunction caused by stress.
WIPI2	Encodes a protein involved in autophagy initiation, acting as a marker for autophagosome formation.
SQSTM1	Encodes p62, a protein involved in autophagy , selective degradation of ubiquitinated proteins and regulates activation of the nuclear factor kappa-B (NF-kB) signaling pathway.
WDR45	Encodes a WD repeat-containing protein involved in autophagy regulation, particularly in the context of neurodegenerative diseases.
GBA1	Encodes glucocerebrosidase which is a lysosomal membrane protein involved in lipid metabolism. Mutations are associated with Gaucher's disease and Parkinson's disease.
UCHL1	Encodes a ubiquitin carboxyl-terminal hydrolase involved in protein degradation pathways and neurodegenerative diseases.
RETREG3	Encodes a protein facilitates endoplasmic reticulum-autophagosome adaptor activity and plays a role in organizing the tubular network of the endoplasmic reticulum.
ZFYVE1	Encodes a protein involved in endosomal trafficking and regulation of cellular signaling pathways.
PHF23	Encodes a protein plays a role in inhibiting autophagosome assembly and maturation while promoting protein ubiquitination.
HTRA2	Encodes a mitochondrial serine protease involved in apoptosis and mitochondrial quality control.
CTSD	Encodes a member of the A1 family of peptidases, a lysosomal aspartic protease involved in protein degradation and cellular homeostasis.
RETREG2	Similar to RETREG3, encodes a protein which is an endoplasmic reticulum (ER)-anchored regulator of autophagy that remains inactive under normal conditions but becomes activated in response to cellular stress.
SMCR8	Encodes a protein involved in TOR signaling and autophagy regulation.
PLEKHM1	Encodes a protein involved in autophagy and endosomal trafficking, particularly in the context of lysosomal function.
ATG2A	Encodes a protein involved in autophagosome formation and autophagy regulation.
MAP1LC3B2	Encodes a variant of the LC3 protein involved in autophagy, acting as a marker for autophagosomes.
ARFIP2	Encodes a protein plays a role in organizing the actin cytoskeleton, regulating mitophagy, and directing protein localization to the phagophore assembly site.
ATP6V1C2	Encodes Part of the V-type ATPase complex, essential for maintaining cellular pH homeostasis.
ULK1 (ATG1)	Encodes a serine/threonine kinase involved in autophagy initiation and regulation.
RUBCN	Encodes rubicon, a protein involved in autophagy regulation and endosomal trafficking.
TP53INP1	Encodes a protein involved in regulating autophagy and cellular stress responses, potentially linked to cancer.
RETREG1	Encodes a protein involved in endosomal trafficking and potentially in neurodegenerative diseases.
SESN2	Encodes sestrin-2, a protein involved in regulating the mTORC1 signaling pathway negatively.
HIF1A	Encodes the alpha subunit of transcription factor hypoxia-inducible factor-1 (HIF-1) involved in hypoxia response and cellular adaptation to low oxygen conditions.
PAFAH1B2	Encodes a protein involved in lipid metabolism.
ATP6V0E1	Encodes a protein which is a part of the V-type ATPase complex, essential for maintaining cellular pH homeostasis.
ATP6V1C1	Encodes a protein which is a part of the V-type ATPase complex, involved in proton transport and cellular homeostasis.

VTI1A	Encodes a protein involved in vesicular transport and membrane trafficking.
BNIP3L	Encodes a protein involved in mitochondrial quality control and autophagy regulation.
ILRUN	Encodes a protein involved in immune responses and functions as an inhibitor of antiviral and proinflammatory cytokine transcription.
TGFB1	Encodes transforming growth factor-beta 1, a cytokine involved in cell growth, differentiation, and extracellular matrix production.
NPC1	Encodes Niemann-Pick C1 protein which locates in the limiting membrane of endosomes and lysosomes, where it facilitates intracellular cholesterol trafficking by binding cholesterol to its N-terminal domain.

References

1. Chen S, Einspanier R, Schoen J. Transepithelial electrical resistance (TEER): a functional parameter to monitor the quality of oviduct epithelial cells cultured on filter supports. Histochemistry and Cell Biology 2015;144:509-515.
2. Kurihara K. Umami the Fifth Basic Taste: History of Studies on Receptor Mechanisms and Role as a Food Flavor. BioMed Research International 2015;2015:189402.
3. 6 Everyday Foods That Contain MSG Available at:

   https://www.ecowatch.com/foods-containing-msg.html

   Accessed 12 Jan, 2022
4. What Is Disodium Guanylate, and Is It Safe? Available at:

   https://www.healthline.com/nutrition/disodium-guanylate#which-foods-contain-it

   Accessed 23 March, 2020

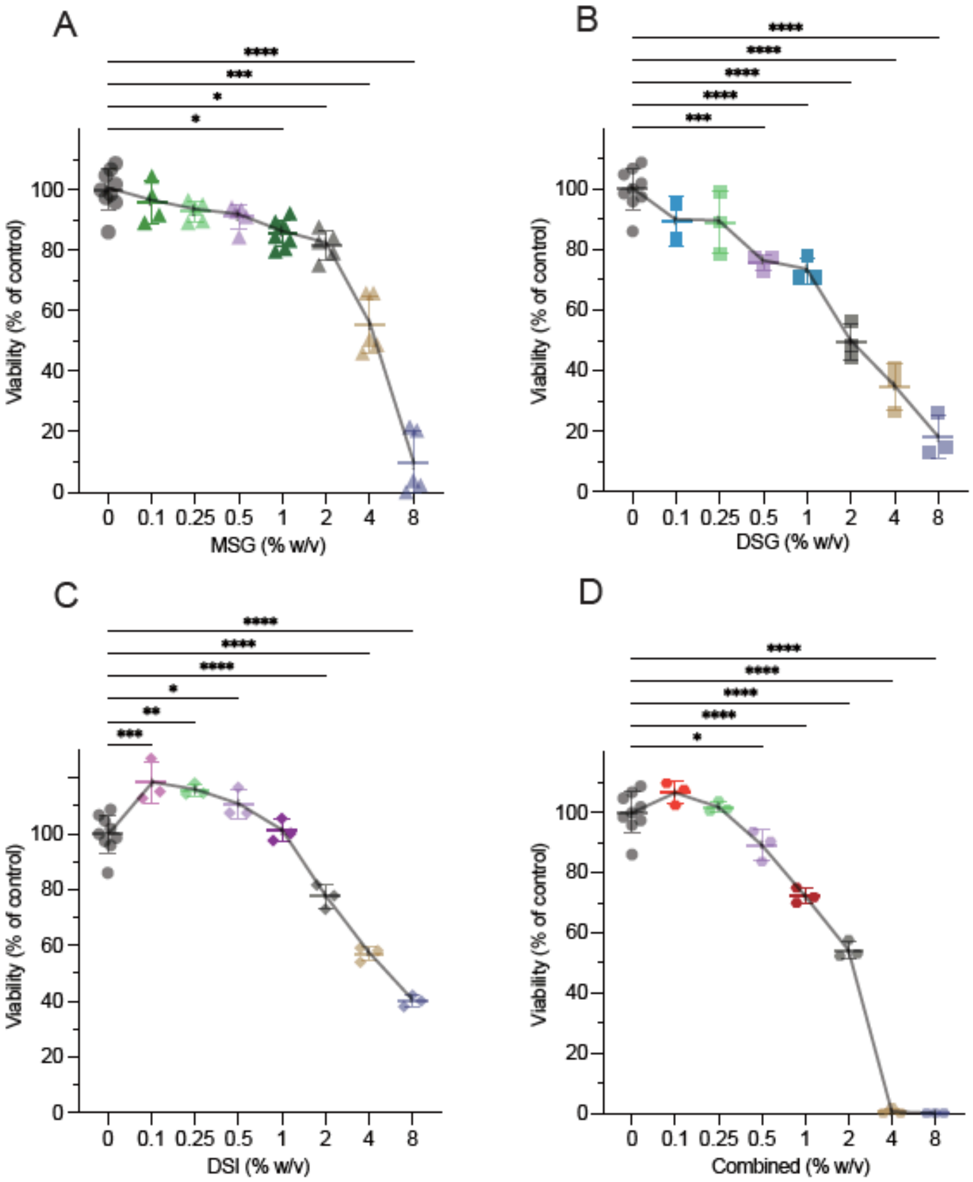

Supplementary Figure 1. The viability of monolayer Caco-2 cells after exposure to different concentrations of MSG, DSG, DSI and their combined effect. The MTT assay quantifies relative cell viability as a percentage of the untreated control after 24 hours of exposure to a series of concentrations in MSG, DSG, DSI and their combined treatment. The results indicated that these compounds exhibit dose-dependent cytotoxicity, with increasing concentrations leading to greater toxic effects on cells. Statistical significance is indicated by asterisks (*p<0.05, **p<0.01, ***p<0.001, ****p<0.0001 by one-way ANOVA). Abbreviations: MSG, monosodium glutamate; DSG, disodium guanylate; DSI, disodium inosinate.

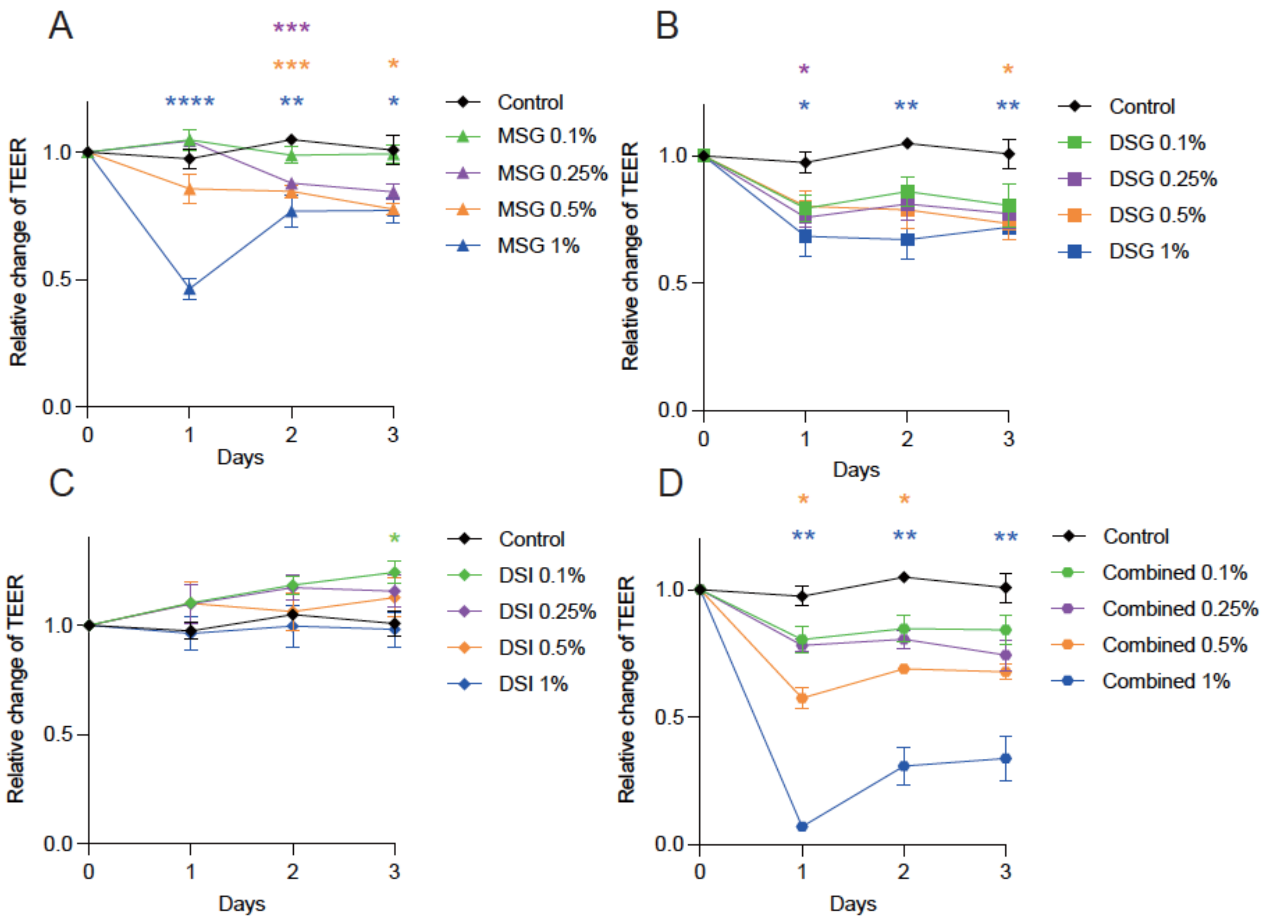

Supplementary Figure 2. The change of TEER values caused by different concentrations of MSG, DSG, DSI and their combined effect. Exposure to 1% MSG, 1% DSG, 1% and 0.5% combined compounds significantly reduced TEER after one day. Although a slight increase was observed on the second day exposed to 1% MSG and 1% combined effect, TEER remained markedly lower overall. Exposure to 0.5% and 0.25% MSG led to a significant reduction in TEER values by the second day. TEER significantly increased after three-day exposure to 0.1% DSI. Line graphs represent mean ± SD, and statistical significance is indicated by asterisks (*p<0.05, **p<0.01, ***p<0.001, ****p<0.0001 by RM two-way ANOVA). Abbreviation: MSG, monosodium glutamate; DSG, disodium guanylate; DSI, disodium inosinate; TEER, transepithelial electrical resistance.

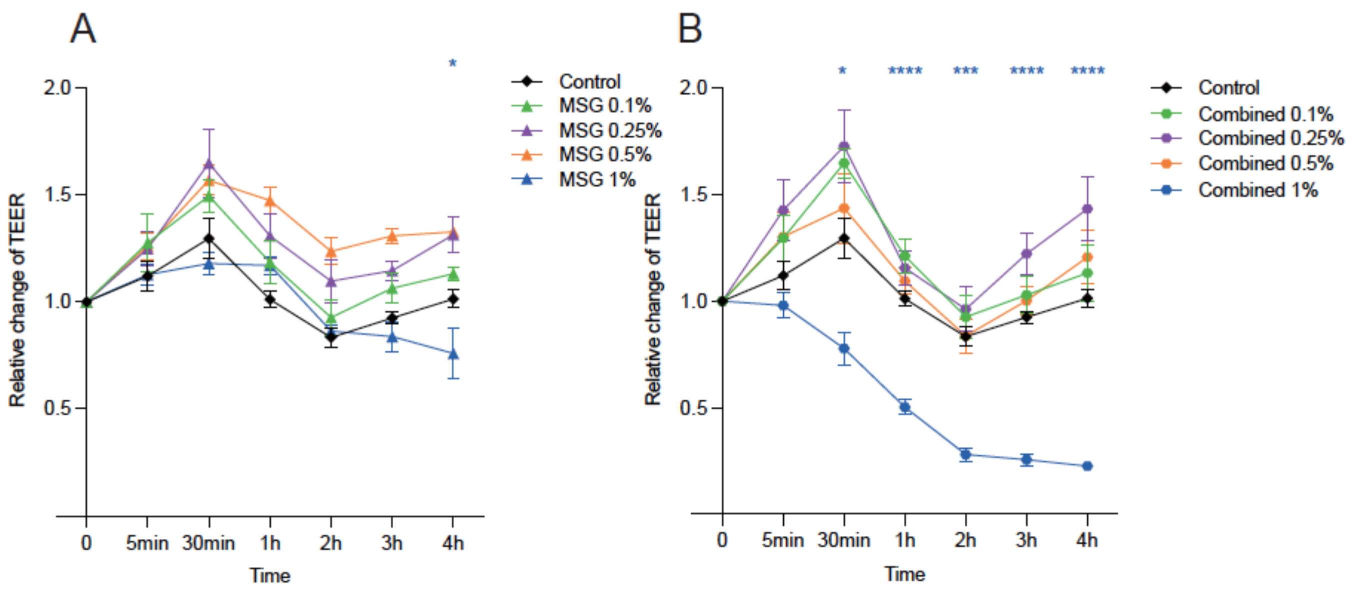

Supplementary Figure 3. The effect of the MSG and comibined on transepithelial resistance (TEER) at short time periods in Caco-2 gut-on-a-chips. A, B, TEER was measured at baseline, 5 min, 30 min and hourly during the first four hours for different concentratios of MSG and the combined treatment. Data are presented as means ± SDs (n=4 for all concentrations).

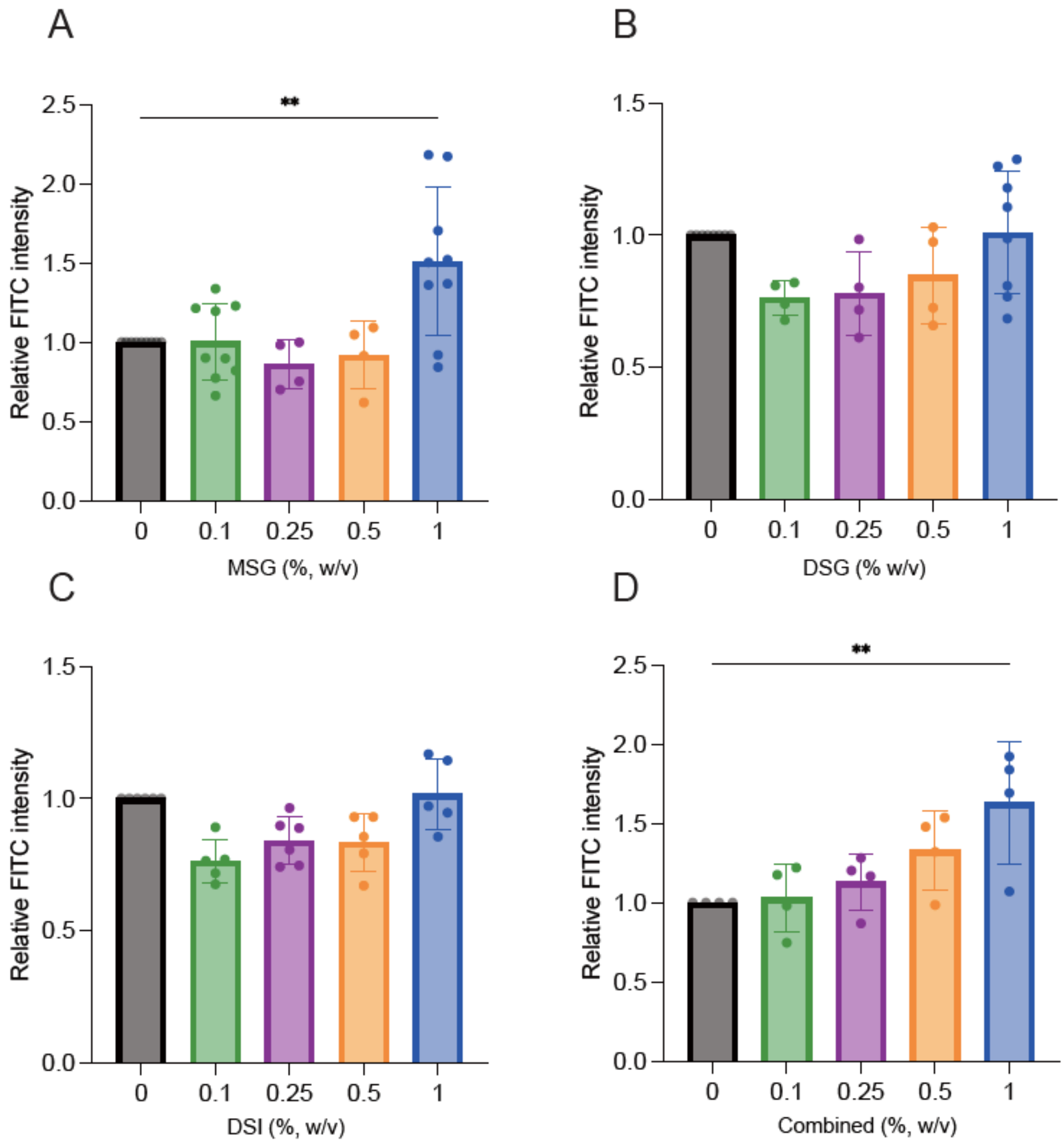

Supplementary Figure 4. The effects of MSG, DSG, DSI and their combined effects on PF were assessed across multiple concentrations. 1% MSG and 1% combined compounds significantly increased PF after three-day exposure. Bars represent mean ± SD, and statistical significance is indicated by asterisks (*$p<0.05$, **$p<0.01$ by one-way ANOVA). Abbreviation: MSG, monosodium glutamate; DSG, disodium guanylate; DSI, disodium inosinate; PF, paracellular flux.

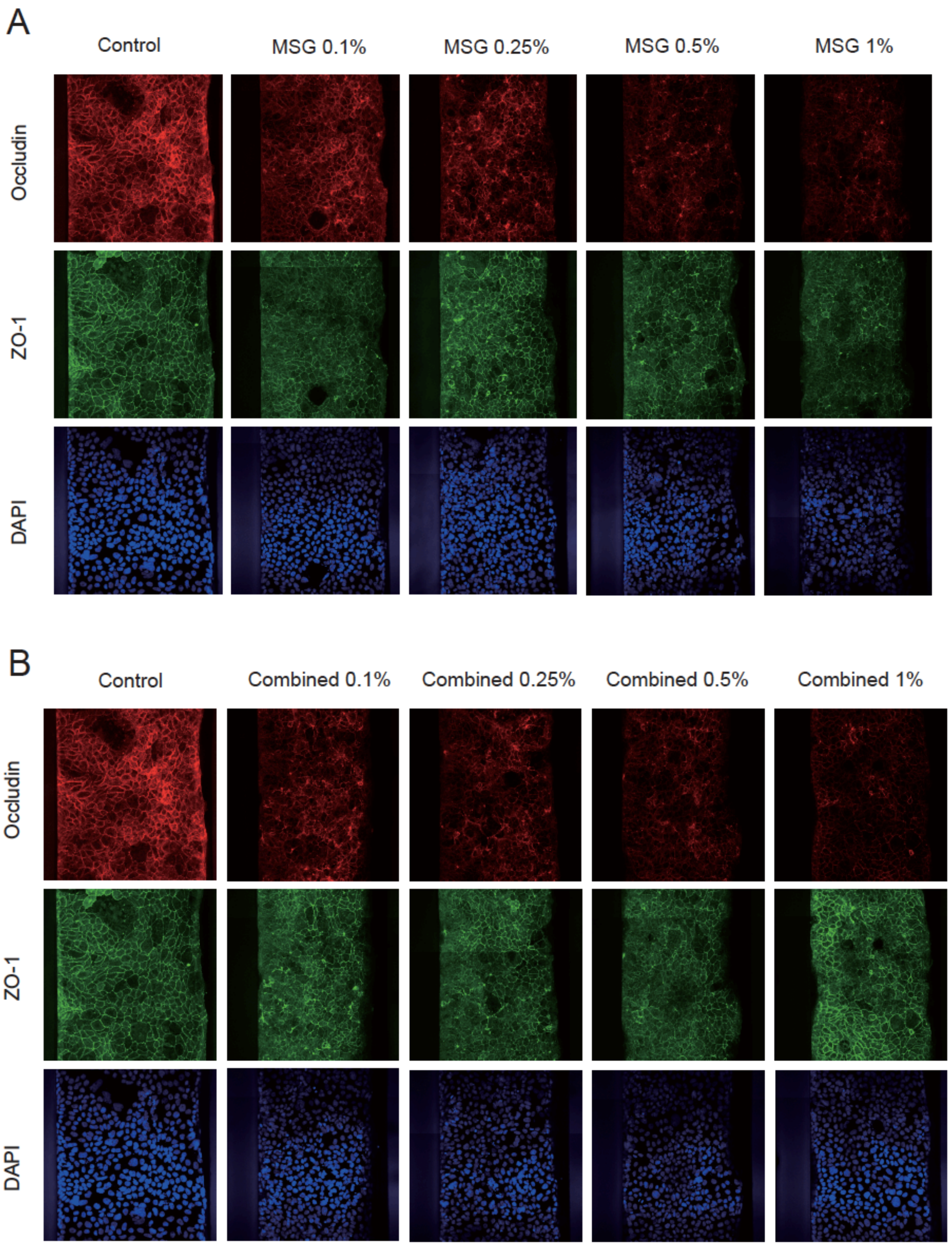

Supplementary Figure 5. Immunofluorescence staining of occludin and DAPI in cells exposed to MSG and combined at different concentrations for 72 h. A representative image of four different stainings is shown.

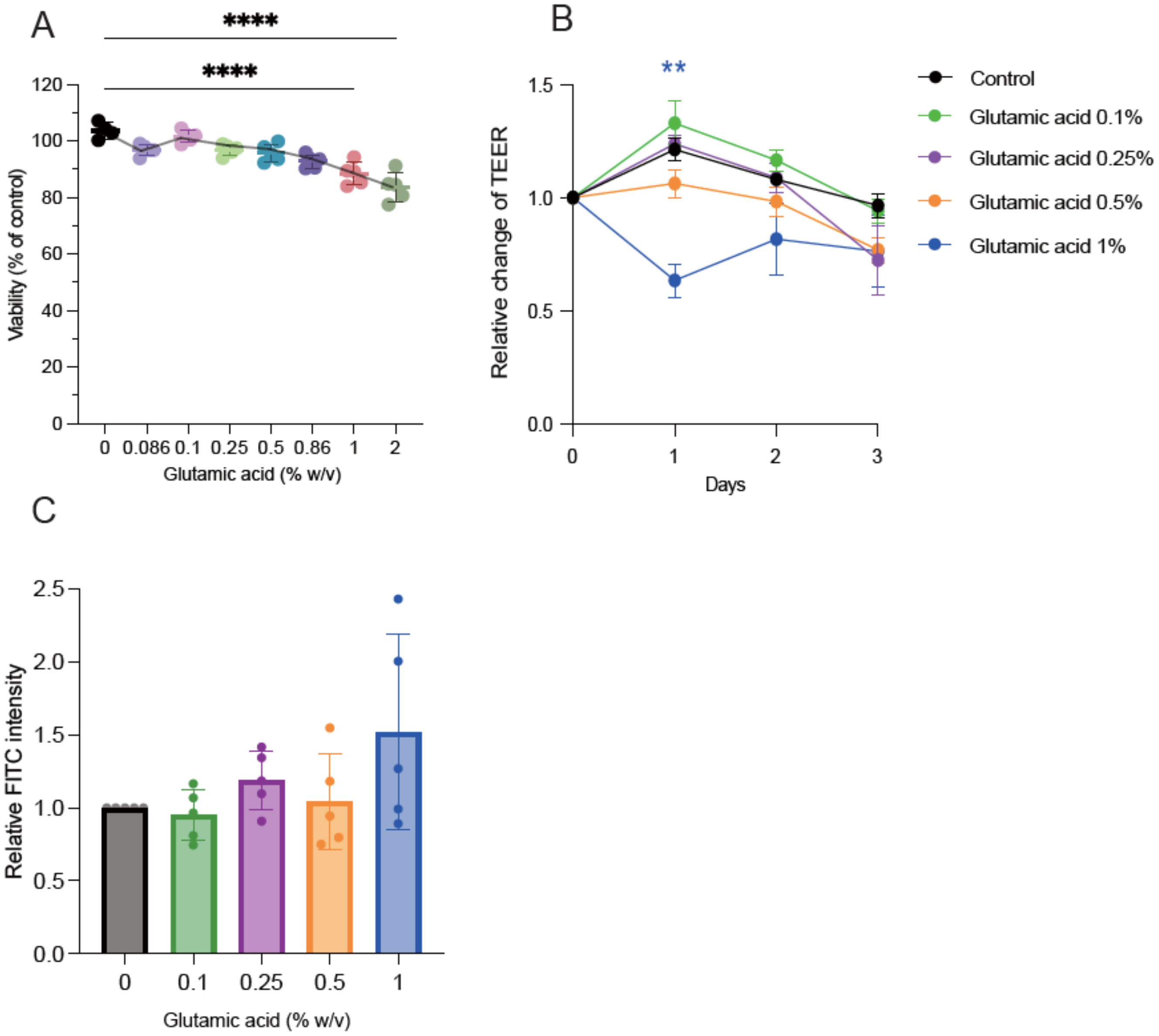

Supplementary Figure 6. Effects of glutamic acid on cell viability, TEER values, and PF values. (A) MTT assay showing cell viability after exposure to varying concentrations of glutamic acid. Cytotoxicity was observed at concentrations of 1% and higher. Statistical significance is indicated by asterisks (****p<0.0001 by one-way ANOVA). (B) TEER significantly decreased after one-day exposure to 1% glutamic acid, indicating compromised barrier integrity. Line graphs represent mean ± SD, and statistical significance is indicated by asterisks (**p<0.01, by RM two-way ANOVA). (C) PF showed a tendency to increase after three-day exposure to 1% glutamic acid. Bars represent mean ± SD Abbreviation: TEER, transepithelial electrical resistance. PF, paracellular flux.
